# Material properties of evolutionary diverse spider silks described by variation in a single structural parameter

**DOI:** 10.1038/srep18991

**Published:** 2016-01-12

**Authors:** Rodrigo Madurga, Gustavo R. Plaza, Todd A. Blackledge, Gustavo.V. Guinea, Manuel Elices, José Pérez-Rigueiro

**Affiliations:** 1 Centro de Tecnología Biomédica. Universidad Politécnica de Madrid. 28223 Pozuelo de Alarcón (Madrid). Spain; 2 Departamento de Ciencia de Materiales. ETSI Caminos, Canales y Puertos. Universidad Politécnica de Madrid. 28040. Madrid. Spain; 3 Department of Biology and Integrated Bioscience Program. The University of Akron, Akron, OH44325-3908. USA

## Abstract

Spider major ampullate gland silks (MAS) vary greatly in material properties among species but, this variation is shown here to be confined to evolutionary shifts along a single universal performance trajectory. This reveals an underlying design principle that is maintained across large changes in both spider ecology and silk chemistry. Persistence of this design principle becomes apparent after the material properties are defined relative to the true alignment parameter, which describes the orientation and stretching of the protein chains in the silk fiber. Our results show that the mechanical behavior of all Entelegynae major ampullate silk fibers, under any conditions, are described by this single parameter that connects the sequential action of three deformation micromechanisms during stretching: stressing of protein-protein hydrogen bonds, rotation of the β-nanocrystals and growth of the ordered fraction. Conservation of these traits for over 230 million years is an indication of the optimal design of the material and gives valuable clues for the production of biomimetic counterparts based on major ampullate spider silk.

## Variability in spider silk

Major ampullate (MA) silk plays a conspicuous role in the construction of webs and safety lines for most of the world’s 45000 species of spiders^1^. The contribution of MA silk to the success of spiders stems from its exceptional tensile strength and extensibility that combine to provide toughness that exceeds almost all known materials^2^,^3^. The extreme variability of MAS fibers across species and even among individuals of the same species^4^,^5^ is not surprising given the diverse ecological niches occupied by these spiders and the variety of biological functions for the silk itself.

**Figure 1:**
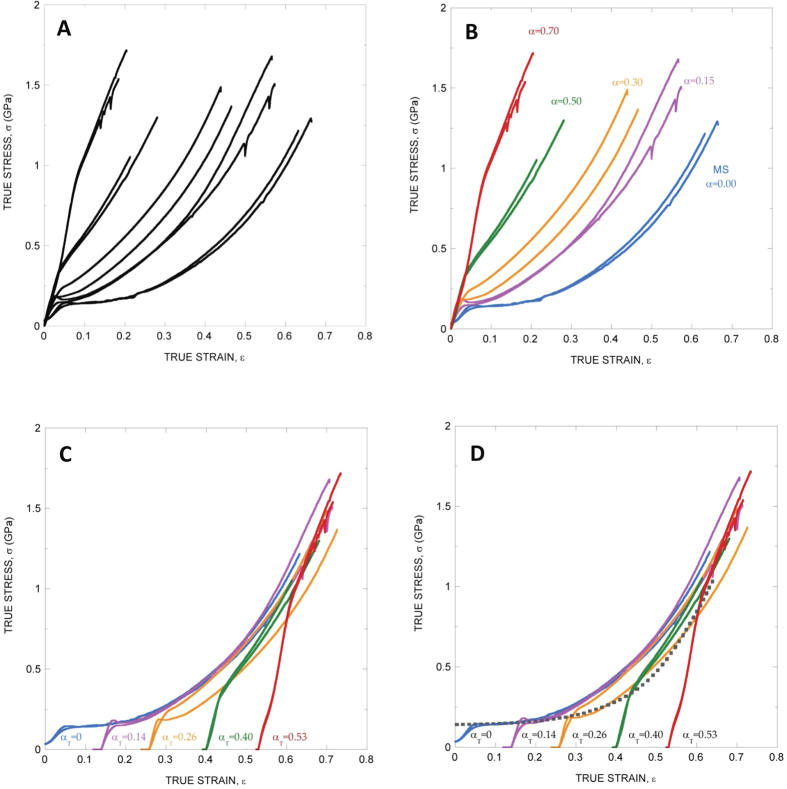
Tensile properties of *Nephila inaurata* MAS. (**A**) Range of tensile properties of *N. inaurata* MAS expressed as true stress-true strain curves. (**B**) The use of the alignment parameter, α, allows classifying the full range of true stress-true strain curves measured from *N. inaurata* MAS. (**C**) The use of the true alignment parameter, α_T_, allows defining the overall tensile behavior of *N. inaurata* MAS in terms of true stress-true strain curves. Each curve is displaced along the true strain axis (X axis) by α_T_ taken the MS curve (α_T_ = 0) as reference. (**D**) Same as in (**C**) but including the true stress-true strain curve of *N. inaurata* MAS tested in water (discontinuous line) and displaced along the true stress axis (Y axis). The value of the displacement is such that the true stress of the displaced curve at the origin (ε = 0) concurs with the yield stress of the MS fiber tested in air.

Figure 1A illustrates this variability by showing the range of tensile properties of MAS fibers spun by a single *Nephila inaurata* specimen. Comparable variability is seen when comparing fibers spun by different species despite carefully controlling the environmental conditions under which spinning takes place, and even measuring the forces exerted on the fiber during the silking process^4^,^6^,^7^,^8^. This variability is supposed to originate at different levels ranging from variation among species in the sequences of silk genes^9^ and differential gene expression^10^, to physiological tuning by individual spiders during spinning^8^ and might play a significant role in the evolutionary success of the group.

However, the low reproducibility found in MAS fibers presents a challenge to understanding the origin of spider silk’s high performance in evolutionary as well as in microstructural terms, and severely limits its application as a biomimetic material^2^. Consequently, significant efforts were devoted to understanding the presumed multifactorial origin of mechanical variability of MAS and to develop new procedures that allowed obtaining silk fibers with reproducible properties^11^,^12^.

Many of these attempts took advantage from the existence of a ground state characteristic of each individual species to which the material can return independently from its previous loading history^13^. It was found that any fiber could be imparted any of the stress-strain curves accessible to a given species by following a procedure named as wet-stretching^13^. Wet-stretching consists of taking the fiber to its ground state through supercontraction, and stretching the supercontracted fiber in water. Stretching is quantified by the alignment parameter, α, defined as the ratio between the increment of length of the fiber after stretching and the initial length of the supercontracted fiber^13^. The whole range of stress-strain curves of MAS spun by a single species can be classified with the definition of the alignment parameter, α, as illustrated in Fig. 1B, and the usage of this parameter allows even the comparison of fibers spun by different species^14^,^15^.

## True alignment parameter

As shown below and illustrated in Fig. 1C, an even better control of the variability of MAS spun by a single species is obtained by modifying the original procedure in two aspects: (i) tensile behaviour is represented in terms of true stress-true strain curves (the original procedure based on the alignment parameter, α, was applied to engineering magnitudes), and (ii) a new true alignment parameter, α_T_, is introduced (see Methods for definition). The true alignment parameter, α_T_, is also a measurement of the elongation of the fiber with respect to its ground state. However, usage of α_T_ is advantageous since it does not only allow the classification of the stress-strain curves, but it is endowed with a significant predicting capability, as explained below.

The predictive ability of α_T_ is clearly shown by the comparison of the post-yield tensile behaviour of MAS fibers. As shown in Fig. 1C when the true stress-true strain curves are displaced along the X (true strain) axis a value equal to the true alignment parameter, concurrence of the curves in the post-yield region is found to be better than 85% (Supplementary Material). The predicting ability of α_T_ can be extended if the true stress-true strain curve of the maximum supercontracted (MS) fiber tested in water is considered. As shown in Fig. 1D the yield points of all curves are accurately predicted by the alignment parameter when the true stress-true strain curve of the MS fiber tested in water is displaced up along the true stress axis (Y-axis), so that the initial value of true stress concurs with the yield stress of the MS fiber (α_T_ = 0.0) tested in air. (yield stress R^2^ = 0.95; yield strain R^2^ = 0.97; maximum relative error between the experimental values of yield stress and the estimated value ~8%).

The displacement along the Y direction of the MS curve tested in water is justified by the simple microstructural model suggested in ref. 16. It is assumed that the behaviour of a silk fiber can be decomposed in two basic deformational mechanisms acting in parallel: entropic chains formed by the silk proteins plus a hydrogen bonding network that sets when the fiber is not in a wet environment. In this regard, the value of the Y-displacement at ε = 0 takes into account the presence of the hydrogen bonds in dry fibers, that are absent when the fiber is immersed in water. The alignment parameter then accurately predicts variation in fiber yield because the contribution of hydrogen bonding is independent of chain orientation in dry silk. The values of the yield stress and yield strain of fibers with different values of α_T_ are shown in Table 1.

Finally, the true alignment parameter can be correlated with the values of the elastic modulus (defined as the initial slope of the true stress-true strain curve) as also shown in Table 1. It is found that the fibers in the MS state (α_T_ = 0.0) show a low value of E ~3 GPa, which increases up to a value of ~8 GPa for α_T_ = 0.14, reaching a final value of ~11 GPa for α_T_ = 0.53. These values are consistent with the model since the increase in the initial slope with increasing α_T_ is explained as the result of the combined effect of a –presumably– constant contribution from hydrogen bonds (close to 3 GPa) and the rising stiffness of the protein chains, represented by the slope of the MS fiber tested in water.

## General validity of the true alignment parameter in the Entelegynae lineage

**Figure 2:**
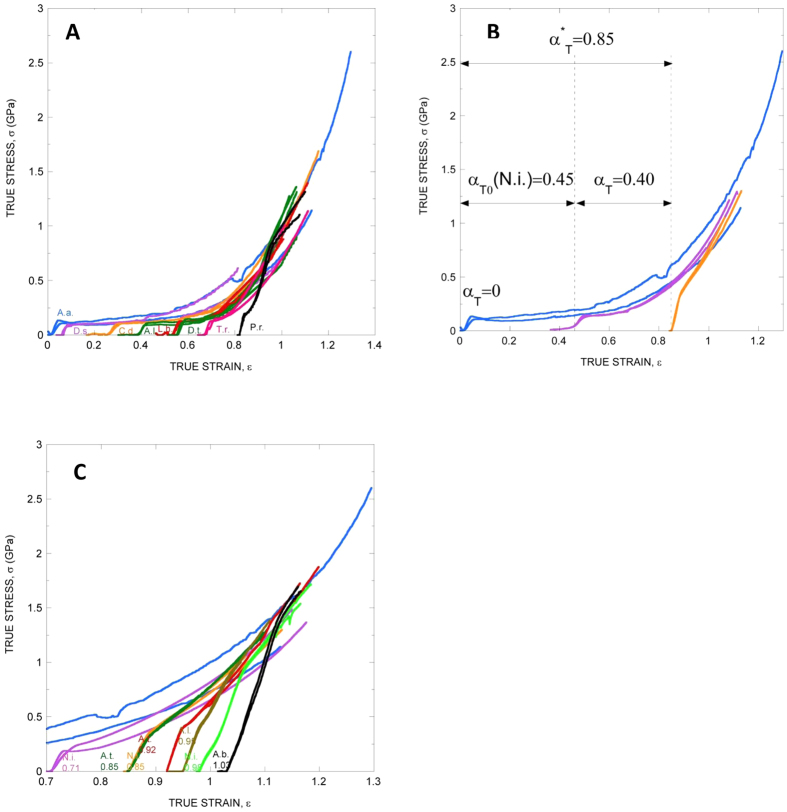
Tensile properties of MAS spun by representatives of the Entelegynae group. (**A**) Concurring tensile behavior of MAS spun by Entelegynae spiders after maximum supercontraction. *Argiope aurantia* maximum supercontracted MAS is used as reference. The interspecific true alignment parameter, α_Τ0_, is defined as the true strain at zero true stress of the curves of any species. A.a.: *Argiope aurantia*, D.s.: *Deinopis spinosa*, C.d: *Caerostris darwini*, A.l.: *Argiope lobata*, L.h.: *Latrodectus hesperus*, D.t.: *Dolomedes tenebrosus*, T.r.: *Tengella radiata*, P.r.: *Phidippus regius*. (**B**) Definition of α^*^_T_ in terms of α_T0_ and α_T_. α_Τ0_(N.i.) labels the interspecific alignment parameter of the MS state of *N. inaurata*. Also represented is the value of α^*^_T_ for *N. inaurata* fibers with a value of the true alignment parameter of α_T_ = 0.4 (Fig. 1C). The value of the interspecific alignment parameter is calculated as α^*^_T_ = α_Τ0_(N.i.) + α_T_. (**C**) Concurring tensile behavior of stretched MAS. The general interspecific alignment parameter, α^*^_T_, is provided below the species identification defined as α^*^_T_ = α_Τ0_(MS) + α_T_. N.i.: *Nephila inaurata*, A.t.: *Argiope trifasciata*, A.l.: *Argiope lobata*, A.b.: *Argiope bruennichi*.

The ability to encapsulate the tensile behaviour of MAS fibers spun by two species (see the results on *Argiope trifasciata* MAS in the Supplementary Material) that diverged over 120 Mya^14^ suggested developing a similar procedure applicable across the Entelegynae lineage. The observation that the post-yield behaviour of silk spun by species showing low extensibility is nearly identical to the performance of silk from species with higher extensibility after stretching (Fig. 2A), suggested generalizing the procedure across the Entelegynae lineage. An interspecific alignment parameter, α_Τ0_, is obtained by comparing the MS true stress-true strain curve of any Entelegynae species with that of MS *Argiope aurantia* MAS. MS *A. aurantia* was used as a reference, since these fibers show the largest strain at breaking of all tested silks^15^. In order to determine the value of α_Τ0_ of any species, the MS curve is displaced along the true strain axis (X-axis) until the true stresses of the curve and that of MS *A. aurantia* concur. Thus, the average curve of a given species in this example goes through the point (ε = 0.94; σ = 0.75 GPa), although the results are relatively independent from the exact point as long as it corresponds to a large value of post-yield strain. The eight species shown in Fig. 2A vary in their post-yield behaviour by less than 20% (Supplementary data). The corresponding values of the interspecific alignment parameter, α_Τ0_, of the MS MAS silks are indicated in Table 2.

The α_T_ and α_Τ0_ combine in a new general interspecific alignment parameter, α^*^_T_ = α_Τ0_ + α_T_, as illustrated in Fig. 2B, that predicts the behaviour of any MAS fiber. In this context, the overall behaviour of any given fiber, as described by α^*^_T_, is broken down into two contributions: α_T0_, which reflects the chemical differences between MAS spun by different species and α_T_, which reflects differences between the fibers deriving from immediate spinning conditions. The predicting ability of α^*^_T_ is illustrated in Fig. 2C, where the true stress-true strain curves of MAS fibers spun by different species and with different values of α_T_ are shown to concur in their post-yield behaviour (maximum relative error between the experimental values and the reference value taken from MS *A. aurantia* MAS ~20% as shown in Supplementary Material).

## True alignment parameter and the design principles of spider silk

**Figure 3:**
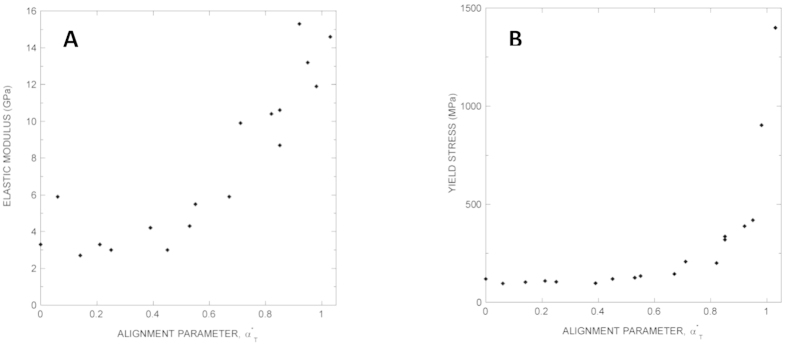
Correlation between elastic modulus (A) and yield stress (B) with the values of the interspecific alignment parameter, α_T0_.

Combining α^*^_T_ with current structure-function models offers a unique opportunity to gain a deeper insight in the design principles of spider silk. MAS is modelled as a composite material in which reinforcing nanocrystals are dispersed in an amorphous matrix^17^,^18^. Reinforcement consists of poly-alanine β-nanocrystals^19^, whose size is tightly controlled to increase toughness^20^,^21^. In turn, the amorphous matrix is represented as a double lattice of hydrogen bonds and elastomeric chains^22^,^23^. The initial behaviour of the fibers is controlled by the deformation of the hydrogen bonds and the stiffening effect of β-nanocrystals and yielding occurs when the hydrogen bonds start to break^16^. Figure 3A shows that the elastic modulus presents an overall dependence with α^*^_T_ that fits to a second order polynomial (R^2^ = 0.95). This dependence of the elastic modulus with α^*^_T_ can be justified as a consequence of the increased alignment of the protein chains with the fiber axis in the rotated nanocrystals.

After yielding the proteins undergo conformational changes associated with the local rotation of the β-nanocrystals^24^,^25^, whose shape does not vary during this process. Figure 3B shows that yield stress is initially invariant with α^*^_T_ but starts increasing after α^*^_T_ ~ 0.6. This dependence of the yield stress with α^*^_T_ is consistent with the assumption that the rotation of the nanocrystals proceeds locally at approximately constant stress, until the alignment of the nanocrystals reaches its maximum. This value can be estimated as α^*^_T_ ~ 0.6 (α ~ 0.5) from *Argiope trifasciata* MAS^26^.

A new deformation micromechanism acts upon completing the rotation of the nanocrystals^20^,^27^. This mechanism consists of the stretching of the proteins and their alignment with the fiber axis^26^,^28^,^29^, and it is accompanied by an increase of the crystalline fraction^26^. The monotonous increase in yield stress at α^*^_T_ in excess of α^*^_T_ > 0.6 suggests that this is a general mechanism that acts across Entelegynae spiders.

The stretched regions of the proteins, in turn, aggregate leading to an increase in the volume fraction of the crystalline phase^26^. The significant increase of the yield stress at high values of α^*^_T_ is consistent with the increase of the crystalline fraction. In this regard, yielding would not result from the breaking of hydrogen bonds, but instead from the accretion of new lengths of the protein chains to the nanocrystals.

It was proposed that the increase in the crystalline fraction was the result of the appearance of a second crystalline phase different from the polyalanine nanocrystals. Models accounting for the microstructure of this second phase varied from unaggregated β-sheets^28^ to oriented amorphous material^29^ or non-periodic lattice crystals (NPL)^28^,^30^. It was assumed that the nanocrystals of this second crystalline phase differed from polyalanine crystals in the presence of a larger number of defects in their structure and were considered as the result of the statistical matching of motifs of sequence different from polyalanine. However, the existence of conformational changes at the ends of the polyalanine crystals^31^ suggested a close relation between both crystalline phases. In this regard, it was naturally assumed that the second crystalline phase would also maintain the β-pleated conformation^32^. However, no direct results on the nature of this new phase or on its relationship with the sequence were obtained from the characterization of MAS fibers. Analyses performed on Flagelliform silk, a type of silk closely related to MAS but which lacks the –A_n_– motif, suggest that this second phase might be the result of aggregating protein chains with a secondary structure of 3_1_ helices in polyglycine II (PG II) nanocrystals^33^. Both the structure and dynamics of PG II observed in Flagelliform silk are compatible with the experimental data on MAS silk at high values of α^*^_T_.

## True alignment parameter and evolution of the Entelegyae lineage

The striking concurrence of all Entelegynae MAS fibers at high true strain regardless of species or previous loading history suggests that the material was selected very early in its evolutionary history for this trait. Conservation of this post-yield performance for over 230 Mya also supports its critical role in silk ecology. In this regard, since the main distinctive feature of the sequence of MAS in Entelegynae is the abundance of the –GGX– motif 34, the previous discussion suggests that this motif is likely responsible for the tensile behaviour of the fibers at high values of α^*^_T_. This suggestion is also strongly supported by the correlation found between the presence of the –GGX– motif and supercontraction^35^.

However, there seems to be a clear distinction between the Orbiculariae and RTA-clade representatives in terms of α^*^_T_, since the MS curves of the Orbiculariae show values of α^*^_T_ < 0.6. Since the mechanical behaviour at these values of α^*^_T_ is controlled by the rotation of the β-nanocrystals, it can be concluded that this mechanism must have been optimized in this group after its separation from the RTA-clade, and is most likely the appearance of the –GPG– motif in the MaSp2 silk of Orbiculariae^36^,^37^,^38^, which promotes the rotational ability of the β-nanocrystals.

**Figure 4:**
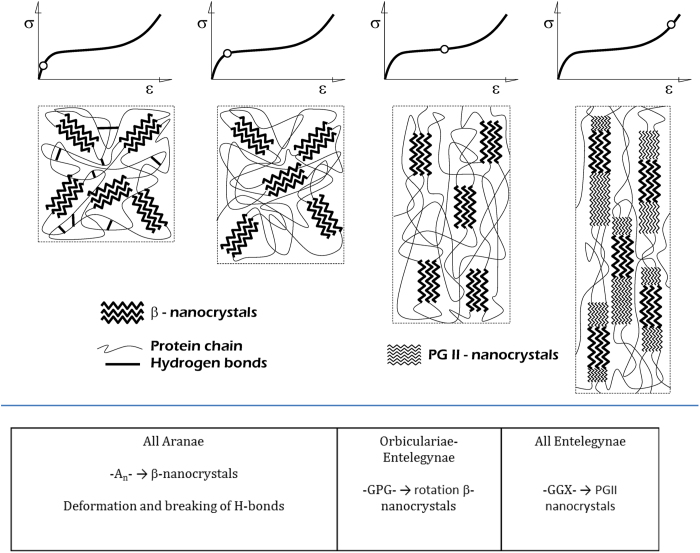
Summary of the microdeformation mechanisms and motifs of sequence of MAS and their relationship with the true alignment parameter, α^*^_T._

Figure 4 is intended to summarize the previous discussion. In this regard, a comprehensive master curve can be built for MAS of Entelegynae spiders, and the behaviour of any species silk accurately predicted by using α^*^_T_ to determine where it lies on that curve and to relate its performance to its molecular structure. This comprehensive curve is the result of the sequential acting of three deformation micromechanisms: stressing and breaking of hydrogen bonds (found in all Aranae MAS), rotation of the β-nanocrystals (mainly found in Orbiculariae) and formation of a second crystalline phase (found in all Entelagynae species). Interestingly, this latter mechanism seems to be at the origin of the group, and is probably related with the abundance of the –GGX– motif. The second mechanism (rotation of β-nanocrystals) is optimized in Orbiculariae MAS and seems to be related with the presence of the –GPG– motif in the sequence.

In conclusion, rather than being a diverse and highly variable set of materials, Entelegynae spider MAS represents an example of extreme conservation of a unique performance trajectory controlling its tensile properties and underlying design principles that has remained essentially stable for over 230 Mya. Rather than opening up new types of performance space, the appearance of novel protein motifs and changes in expression levels of different MaSp genes instead correlate with shifts in MAS silk performance along this single trajectory. This suggests strong action by natural selection not so much to diversify spider silks, but instead to elaborate upon a single archetypal material.

## Methods

### Silk collection

Major ampullate gland silk fibers from representative species of several major families in the Entelegynae were used in this study. Species come from seven families: Araneidae (*Argiope trifasciata*, *Argiope aurantia*, *Argiope lobata*, *Argiope bruennichi*, *Araneus diadematus*, *Caerostris darwini*), Deinopidae (*Deinopis spinosa*), Theriidae (*Latrodectus hesperus*), Pisauridae (*Dolomedes tenebrosus*), Tengellidae (*Tengella radiata*), and Salticidae (*Phidippus regius*).

Silk was collected from the spiders by forced silking and all samples were subjected to a maximum supercontraction process^13^, which consists of an initial step of immersion in water and subsequent drying in air overnight. Maximum supercontraction (MS) requires that the fiber remains slack throughout the whole process as was always assessed before further manipulating the fibers. The length of the fiber after maximum supercontraction is indicated by L_MS_. The tensile behaviour of some samples was modified through a wet stretching process^13^. Briefly, maximum supercontrated samples were stretched while immersed in water up to a predetermined length, L_C_, and allowed to dry overnight. Wet stretching is characterized by the alignment parameter, α, defined as:


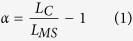


the true alignment parameter, α_T_, is defined from the original definition of alignment parameter, α, as:


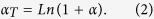


### Tensile testing

20 mm-long samples were tested in an Instron 4411 tensile testing machine at a speed of 0.02 mm/s^−1^ under nominal environmental conditions 23 °C and 40% RH. All samples were tested at the same speed to prevent any differences between the tests that might arise from the time dependent behaviour of the fibers. Loads were measured with a balance Precisa XT 220 (resolution ± 1 μN) and displacement was computed directly from the displacement of the crosshead (resolution ± 10 μm), since the compliance of the fiber is at least 1000 times larger than that of the rest of the experimental setup.

The cross sectional areas were measured from SEM micrographs of samples adjacent to those tensile tested. Five diameters were measured for each micrograph and the area was calculated assuming a circular cross section.

Displacements and forces were converted into true strains, ε, and true stresses, σ, as:


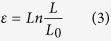



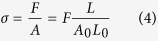


where A_0_ and L_0_ are, respectively, the initial area and length of the sample, and A and L are the instantaneous values of these magnitudes. Instantaneous cross sectional areas were calculated from the measured ones under the hypothesis of constant volume, that was proved for *Argiope trifasciata* major ampullate gland silk fibers^39^.

### Statistical comparisons

To compare similarity of different stress-strain curves we followed Garrido *et al*.^40^ and computed the relative error (RE) as:


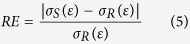


where σ_S_(ε) is the value of the true stress at a given value, ε, of true strain and σ_R_(ε) is the value of the true stress of the reference curve at the same value of true strain.

## Additional Information

**How to cite this article**: Madurga, R. *et al*. Material properties of evolutionary diverse spider silks described by variation in a single structural parameter. *Sci. Rep*. **6**, 18991; doi: 10.1038/srep18991 (2016).

## Supplementary Material

Supplementary Information

## Figures and Tables

**Table 1:** Elastic modulus, (E), yield stress (σ_y_) and yield strain (ε_y_) of *Nephila inaurata* MAS fibers as a function of the true alignment parameter, α_T_.

α_T_	0.00	0.14	0.26	0.40	0.53
E (GPa)	3.0 ± 0.1	8 ± 1	9 ± 2	9.5 ± 0.9	11 ± 1
σ_y_ (MPa)	120 ± 20	140 ± 20	200 ± 10	320 ± 20	920 ± 20
ε_y_	0.035 ± 0.005	0.020 ± 0.004	0.022 ± 0.007	0.032 ± 0.004	0.079 ± 0.001

**Table 2:** True alignment parameter of different Entelegynae species taking the maximum supercontracted state of *Argiope aurantia* MAS as reference.

Species	α_T0_
*Argiope aurantia*	0.00
*Deinopis spinosa*	0.06
*Argiope bruennichi*	0.14
*Argiope trifasciata*	0.21
*Caerostris darwini*	0.25
*Argiope lobata*	0.39
*Nephila inaurata*	0.45
*Latrodectus hesperus*	0.53
*Dolomedes tenebrosus*	0.55
*Tengella radiata*	0.67
*Phidippus regius*	0.82

## References

[b1] Blackledge T. A. . Reconstructing web evolution and spider diversification in the molecular era. Proc. Natl. Acad. Sci. USA 106, 5229–5234 (2009). 19289848 10.1073/pnas.0901377106 PMC2656561

[b2] Heim M., Keerl D. & Scheibel T. Spider silk: from soluble protein to extraordinary fiber. Angew. Chem. Int. Ed Engl. 48, 3584–96 (2009). 19212993 10.1002/anie.200803341

[b3] Cranford S. W., Tarakanova A., Pugno N. M. & Buehler M. J. Nonlinear material behaviour of spider silk yields robust webs. Nature 482, 72–76 (2012). 22297972 10.1038/nature10739

[b4] Work R. W. Dimensions, Birefringences, and Force-Elongation Behavior of Major and Minor Ampullate Silk Fibers from Orb-Web-Spinning Spiders - Effects of Wetting on these Properties. Text. Res. J. 47, 650–662 (1977).

[b5] Madsen B., Shao Z. Z. & Vollrath F. Variability in the mechanical properties of spider silks on three levels: interspecific, intraspecific and intraindividual. Int. J. Biol. Macromol. 24, 301–306 (1999). 10342779 10.1016/s0141-8130(98)00094-4

[b6] Madsen B. & Vollrath F. Mechanics and morphology of silk drawn from anesthetized spiders. Naturwissenschaften 87, 148–153 (2000). 10798202 10.1007/s001140050694

[b7] Vollrath F., Madsen B. & Shao Z. Z. The effect of spinning conditions on the mechanics of a spider’s dragline silk. Proceedings of the Royal Society of London Series B-Biological Sciences 268, 2339–2346 (2001). 10.1098/rspb.2001.1590 PMC1088885 11703874

[b8] Ortlepp C. & Gosline J. Consequences of forced silking. Biomacromolecules 5, 727–731 (2004). 15132653 10.1021/bm034269x

[b9] Rudall K. M. & Kenchington W. Arthropod Silks the Problem of Fibrous Proteins in Animal Tissues. Annu. Rev. Entomol., 73–96 (1971).

[b10] Marhabaie M., Leeper T. C. & Blackledge T. A. Protein Composition Correlates with the Mechanical Properties of Spider (Argiope trifasciata) Dragline Silk. Biomacromolecules 15, 20–29 (2014). 24313814 10.1021/bm401110b

[b11] Perez-Rigueiro J., Elices M. & Guinea G. V. Controlled supercontraction tailors the tensile behaviour of spider silk. Polymer 44, 3733–3736 (2003).

[b12] Liu Y., Shao Z. Z. & Vollrath F. Relationships between supercontraction and mechanical properties of spider silk. Nature Materials 4, 901–905 (2005). 16299506 10.1038/nmat1534

[b13] Guinea G. V., Elices M., Perez-Rigueiro J. & Plaza G. R. Stretching of supercontracted fibers: a link between spinning and the variability of spider silk. J. Exp. Biol. 208, 25–30 (2005). 15601874 10.1242/jeb.01344

[b14] Elices M. . Mechanical Behaviour of Silk during the Evolution of Orb-web Spinning Spiders. Biomacromolecules 10, 1904–1910 (2009). 19505138 10.1021/bm900312c

[b15] Blackledge T. A. . Sequential origin in the high performance properties of orb spider dragline silk. Sci. Rep. 2, 782 (2012). 23110251 10.1038/srep00782 PMC3482764

[b16] Planas J., Guinea G. V. & Elices M. Constitutive model for fiber-reinforced materials with deformable matrices. Physical Review E 76, 041903 (2007). 10.1103/PhysRevE.76.041903 17995022

[b17] Termonia Y. Molecular Modeling of Spider Silk Elasticity. Macromolecules 27, 7378–7381 (1994).

[b18] Du N. . Design of superior spider silk: From nanostructure to mechanical properties. Biophys. J. 91, 4528–4535 (2006). 16950851 10.1529/biophysj.106.089144 PMC1779941

[b19] Warwicker J. O. Comparative Studies of Fibroins .2. Crystal Structures of various Fibroins. J. Mol. Biol. 2, 350–362 (1960). 13783274 10.1016/s0022-2836(60)80046-0

[b20] Keten S., Xu Z. P., Ihle B. & Buehler M. J. Nanoconfinement controls stiffness, strength and mechanical toughness of beta-sheet crystals in silk. Nature Materials 9, 359–367 (2010). 20228820 10.1038/nmat2704

[b21] Nova A., Keten S., Pugno N. M., Redaelli A. & Buehler M. J. Molecular and Nanostructural Mechanisms of Deformation, Strength and Toughness of Spider Silk Fibrils. Nano Letters 10, 2626–2634 (2010). 20518518 10.1021/nl101341w

[b22] Gosline J. M., Denny M. W. & Demont M. E. Spider Silk as Rubber. Nature 309, 551–552 (1984).

[b23] Keten S. & Buehler M. J. Nanostructure and molecular mechanics of spider dragline silk protein assemblies. Journal of the Royal Society Interface 7, 1709–1721 (2010). 10.1098/rsif.2010.0149 PMC2988266 20519206

[b24] Work R. W. & Morosoff N. A Physicochemical Study of the Supercontraction of Spider Major Ampullate Silk Fibers. Text. Res. J. 52, 349–356 (1982).

[b25] Eles P. T. & Michal C. A. Strain dependent local phase transitions observed during controlled supercontraction reveal mechanisms in spider silk. Macromolecules 37, 1342–1345 (2004).

[b26] Plaza G. R. . Relationship between microstructure and mechanical properties in spider silk fibers: two regimes in the microstructural changes. Soft Matter 8, 6015–6026 (2012).

[b27] Guan J., Vollrath F. & Porter D. Two Mechanisms for Supercontraction in Nephila Spider Dragline Silk. Biomacromolecules 12, 4030–4035 (2011). 21951163 10.1021/bm201032v

[b28] Simmons A., Michal C. & Jelinski L. Molecular orientation and two-component nature of the crystalline fraction of spider dragline silk. Science 271, 84 (1996). 8539605 10.1126/science.271.5245.84

[b29] Grubb D. T. & Jelinski L. W. Fiber morphology of spider silk: The effects of tensile deformation. Macromolecules 30, 2860–2867 (1997).

[b30] Thiel B. L. & Viney C. A Nonperiodic Lattice Model for Crystals in Nephila-Clavipes Major Ampullate Silk. MRS Bull 20, 52–56 (1995).

[b31] Paquet-Mercier F., Lefevre T., Auger M. & Pezolet M. Evidence by infrared spectroscopy of the presence of two types of beta-sheets in major ampullate spider silk and silkworm silk. Soft Matter 9, 208–215 (2013).

[b32] Li X., Eles P. T. & Michal C. A. Water Permeability of Spider Dragline Silk. Biomacromolecules 10, 1270–1275 (2009). 19331319 10.1021/bm900103n

[b33] Perea G. B. . Identification and dynamics of polyglycine II nanocrystals in Argiope trifasciata flagelliform silk. Scientific Reports 3, 3061 (2013). 24162473 10.1038/srep03061 PMC3808813

[b34] Gatesy J., Hayashi C., Motriuk D., Woods J. & Lewis R. Extreme diversity, conservation, and convergence of spider silk fibroin sequences. Science 291, 2603–2605 (2001). 11283372 10.1126/science.1057561

[b35] Yang Z. T. . Supercontraction and backbone dynamics in spider silk: C-13 and H-2 NMR studies. J. Am. Chem. Soc. 122, 9019–9025 (2000).

[b36] Blackledge T. A. . How super is supercontraction? Persistent versus cyclic responses to humidity in spider dragline silk. J. Exp. Biol. 212, 1980–1988 (2009). 10.1242/jeb.028944 19525422

[b37] Boutry C. & Blackledge T. A. Evolution of supercontraction in spider silk: structure-function relationship from tarantulas to orb-weavers. J. Exp. Biol. 213, 3505–3514 (2010). 20889831 10.1242/jeb.046110

[b38] Liu Y., Sponner A., Porter D. & Vollrath F. Proline and processing of spider silks. Biomacromolecules 9, 116–121 (2008). 18052126 10.1021/bm700877g

[b39] Guinea G. V., Perez-Rigueiro J., Plaza G. R. & Elices M. Volume constancy during stretching of spider silk. Biomacromolecules 7, 2173–2177 (2006). 16827584 10.1021/bm060138v

[b40] Garrido M. A., Elices M., Viney C. & Perez-Rigueiro J. The variability and interdependence of spider drag line tensile properties. Polymer 43, 4495–4502 (2002).

