# Supplementary material for: Material properties of evolutionary diverse spider silks described by variation in a single structural parameter
Source: Sci Rep. 2016 Jan 12;6:18991. doi: 10.1038/srep18991 (PMC4709512; doi:10.1038/srep18991)
Supplement: Supplementary Information [file srep18991-s1.pdf]

Material properties of evolutionary diverse spider silks described by variation in a single structural parameter

SUPPLEMENTARY MATERIAL

Rodrigo Madurga[1,2], Gustavo R. Plaza[1,2], Todd A. Blackledge[3], Gustavo.V. Guinea[1,2], Manuel Elices[1,2], José Pérez-Rigueiro[1,2]

1. Centro de Tecnología Biomédica. Universidad Politécnica de Madrid. 28223 Pozuelo de Alarcón (Madrid). Spain
2. Departamento de Ciencia de Materiales. ETSI Caminos, Canales y Puertos. Universidad Politécnica de Madrid. 28040. Madrid. Spain
3. Department of Biology and Integrated Bioscience Program. The University of Akron, Akron, OH44325-3908. USA

Main notation and abbreviations

L_{MS}: length of the fiber after maximum supercontraction

MA(S): major ampullate (silk)

MS: maximum supercontraction

RE: relative error

α: alignment parameter

α_T: (intraspecific) true alignment parameter

α_{T0}: (MS) interspecific alignment parameter

α^*_T: (general) interspecific alignment parameter

ε: true strain

σ: true stress

Supplementary Material Figure 1. Tensile properties of *Argiope trifasciata* MAS as a function of the true alignment parameter, α_T. The use of the true alignment parameter, α_T, allows defining the overall tensile behavior of *A. trifasciata* MAS in terms of true stress-true strain curves. Each curve is displaced along the true strain axis (X axis) by α_T taken the MS curve (α_T=0) as reference. The true stress-true strain curve of *A. trifasciata* MAS tested in water and displaced along the true stress axis (discontinuous line) is also shown. The value of the displacement is such that the true stress of the displaced curve at the origin (ε=0) concurs with the yield stress of the MS fiber tested in air.

Quantitative comparison of the stress-strain curves

Prior to computing the relative error, the average true stress-true strain curve of any given condition defined by the species and the true alignment parameter of the fiber, α_T, is calculated as the arithmetic mean of the true stresses of all curves corresponding to that condition for each value of the true strain. A mean curve is calculated following the same procedure for the condition selected as reference (usually the MS curves of *A. aurantia* MAS). In order to increase the region of strains at which the curves overlap, the values of the curves up to a maximum strain of ε=1.2 were extrapolated by fitting the experimental results to a second order polynomial. In all cases, the correlation coefficient of the second order polynomial used to fit the experimental curve was R^2>0.98. The relative error was calculated from the mean curves using equation (4). The results of the relative errors corresponding to Figures 1c, 2a and 2c, and Supplementary Material Figure 1 are presented in Supplementary Material Figures 2a-d.

Supplementary Material Figure 2a. Relative error of the true stress-true strain curves of *Nephila inaurata* MAS shown in Figure 1c.

Relative errors of *N. inaurata* MAS fibers corresponding to different values of the alignment parameter taking the mean curve of the MS condition (α_T=0.0) as reference. Relative errors are shown to decrease with increasing values of true strain. The relative error found between the individual curves of the samples with alignment parameter α_T=0.26, which corresponds to the maximum value of RE of samples with the same value of α_T, is shown for comparison purposes (dashed line).

Supplementary Material Figure 2b. Relative error of the true stress-true strain curves of *Argiope trifasciata* MAS shown in Supplementary Material Figure 1.

Relative errors of *A. trifasciata* MAS fibers corresponding to different values of the alignment parameter taking the mean curve of the MS condition(α_T=0.0) as reference. Relative errors are shown to decrease with increasing values of true strain. The relative error found between the individual curves of the samples with alignment parameter α_T=0.00 (i.e. Maximum supercontracted fibers), which corresponds to the maximum value of RE of samples with the same value of α_T, is shown for comparison purposes (dashed line).

Supplementary Material Figure 2c. Relative error of the true stress-true strain curves of representatives of the Entelegynae MAS in the maximum supercontrated state shown in Figure 2a.

Relative errors of MAS fibers spun by representatives of the Entelegynae whose true stress-true strain curves are shown in Figure 2a taking the mean curve of *Argiope aurantia* MAS in the MS condition (α_{T0}=0.0) as reference. The relative error found between the individual curves of the MS *A. aurantia* samples are shown for comparison purposes (dashed line).

Supplementary Material Figure 2d. Relative error of the true stress-true strain curves of representatives of the Entelegynae MAS with different values of α_T shown in Figure 2c.

Relative errors of MAS fibers spun by representatives of the Entelegynae whose true stress-true strain curves are shown in Figure 2c taking the mean curve of *Argiope aurantia* MAS in the MS condition (α_{T0}=0.0) as reference. The relative error found between the individual curves of the MS *A. aurantia* samples are shown for comparison purposes (dashed line).

Extension of the model to other Araneae lineages (Haplogynae and Mygalomorphae)

As shown by the previous data, the predicting ability of the general true alignment parameter, α^*_T, works nicely when applied to MAS fibers spun by Entelegynae spiders. Entelegynae species represent over 75% of all extant spiders, a clear indication of its evolutionary success, despite its appearance some 230 Mya ago does correspond to just

half of the history of the Opisthothelae group dating back to 392 Mya. In this context, it is worth analyzing whether a similar construction might encompass the silk spun by the other main representatives of the Opisthothelae: Mygalomorphae and Haplogynae.

Mygalomorphae silk is characterized by the presence of the polyalanine motif and the absence of the other motifs found in Araneomorphae (Haplogynae and Entelegynae). The polyalanine motifs form β-nanocrystals with a low degree of orientation with the macroscopic axis of the fiber and do not show relevant supercontraction.

The main motifs found in Haplogynae species are polyalanine and polyalanineglycine. Some –GGX– motifs are found in the sequence but in a much lower proportion than in Entelegynae species. The main microstructural motif of the group seems to be β-poly-glycine-alanine nanocrystals that are highly oriented with the macroscopic axis of the fiber. Silks from this group show low values of supercontraction (if at all present).

Supplementary Material Figure 3 shows the comparison of the true stress-true strain curves of one representative of the Mygalomorphae (*Aphonopelma seemani*), two representatives of the Haplogynae (*Kukulcania hibernalis* and *Scytodes* sp.) and the maximum supercontracted state of *Argiope aurantia* MAS. In parallel with the general procedure, true stress-true strain curves were displaced along the true strain axis (X axis) to check the concurrence of the curves at large values of strains. The results in this case are much less conclusive, since the reduced value of the strain at breaking of the Mygalomorphae and Haplogynae representatives limits the region in which the concurrence of the curves can be checked. However, it is found that the inclusion of Mygalomorphae silk and Haplogynae MAS does not contradict the previous scheme. In particular, Supplementary Material Figure 3 might imply that Mygalomorphae silk presents the rotation of β-nanocrystals as main microdeformation mechanism, while this mechanism would be substituted by the stretching of the chains in Haplogynae.

Supplementary Material Figure 3. Comparison of the silk of Mygalomorphae and Haplogynae representatives with maximum supercontrated *Argiope aurantia* MAS. Tensile properties of *Aphonopelma seemani* (A.s.; Mygalomorphae), and *Kukulcania hibernalis* (K.h.; Haplogynae) and *Scytodes* sp. (S.s.; Haplogynae) compared with maximum supercontrated *A. aurantia* MAS.

Supplementary Material Figure 4. ***Nephila clavipes*** **hanging from its MAS safety line.**

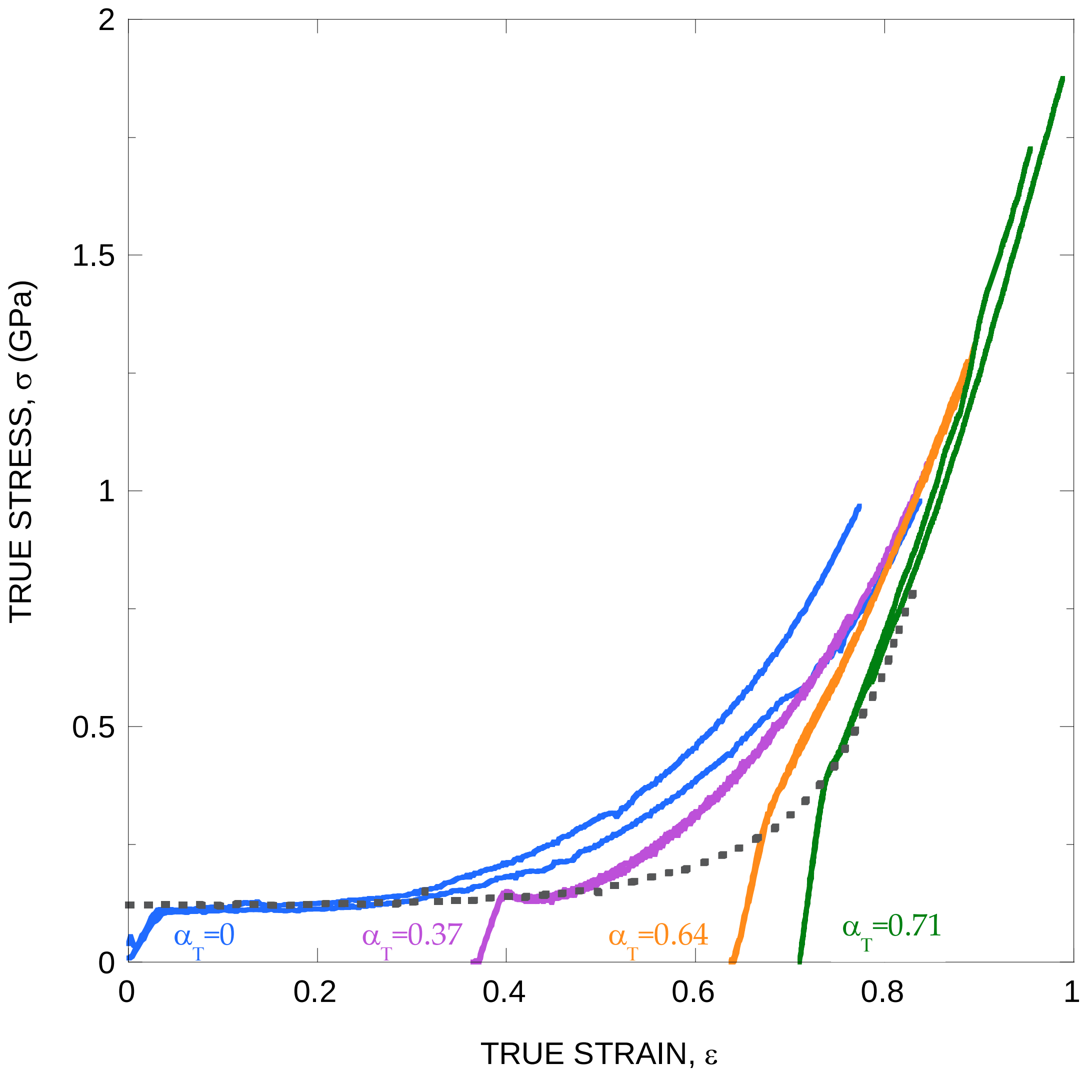

Supplementary Material Figure 1

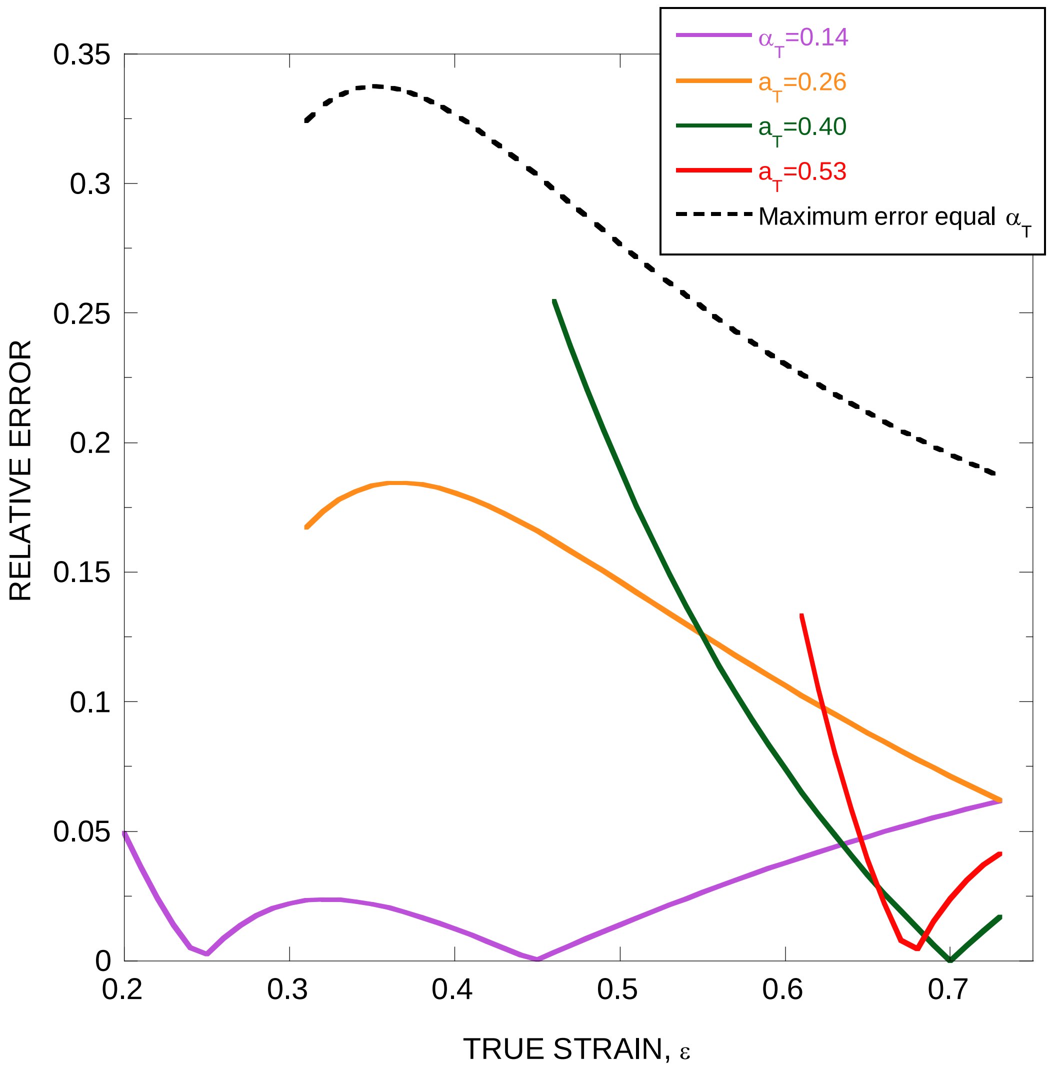

Supplementary Material Figure 2a

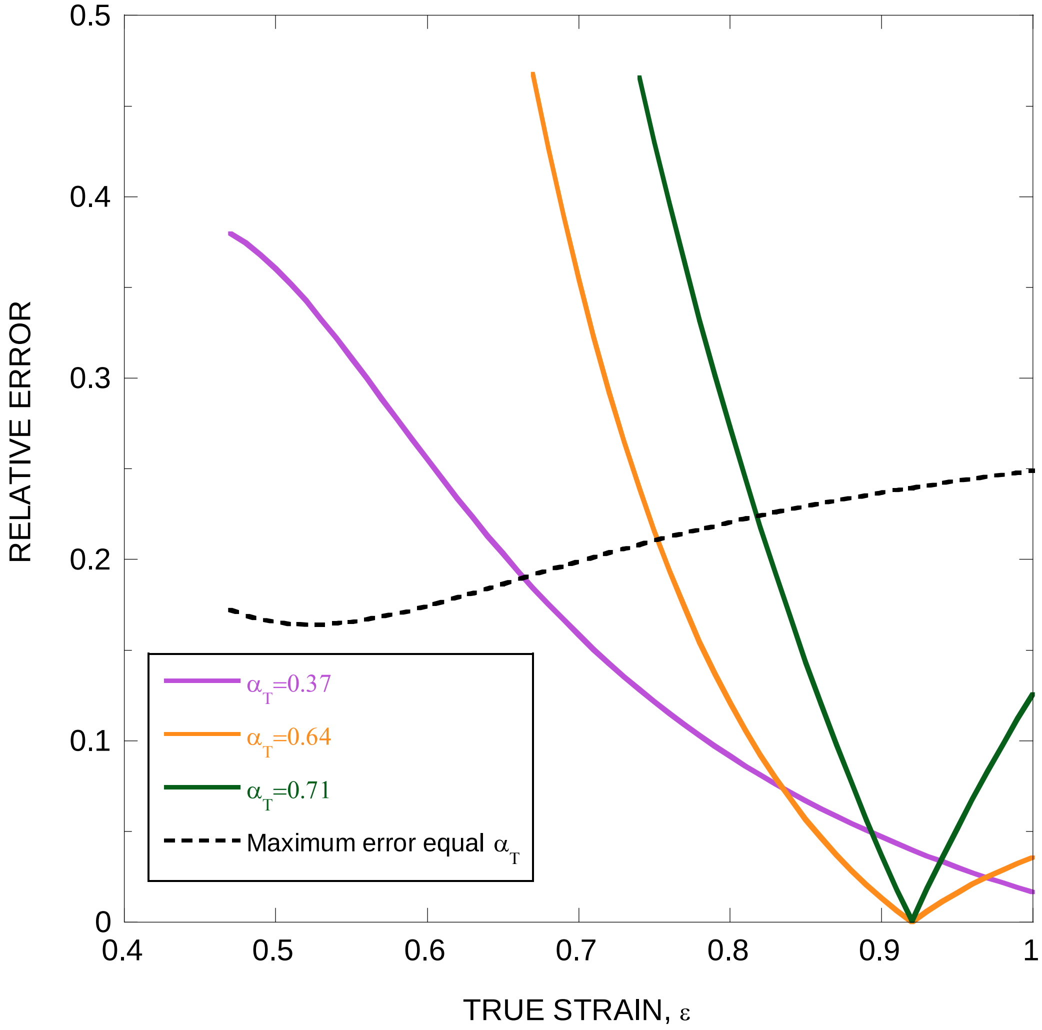

Supplementary Material Figure 2b

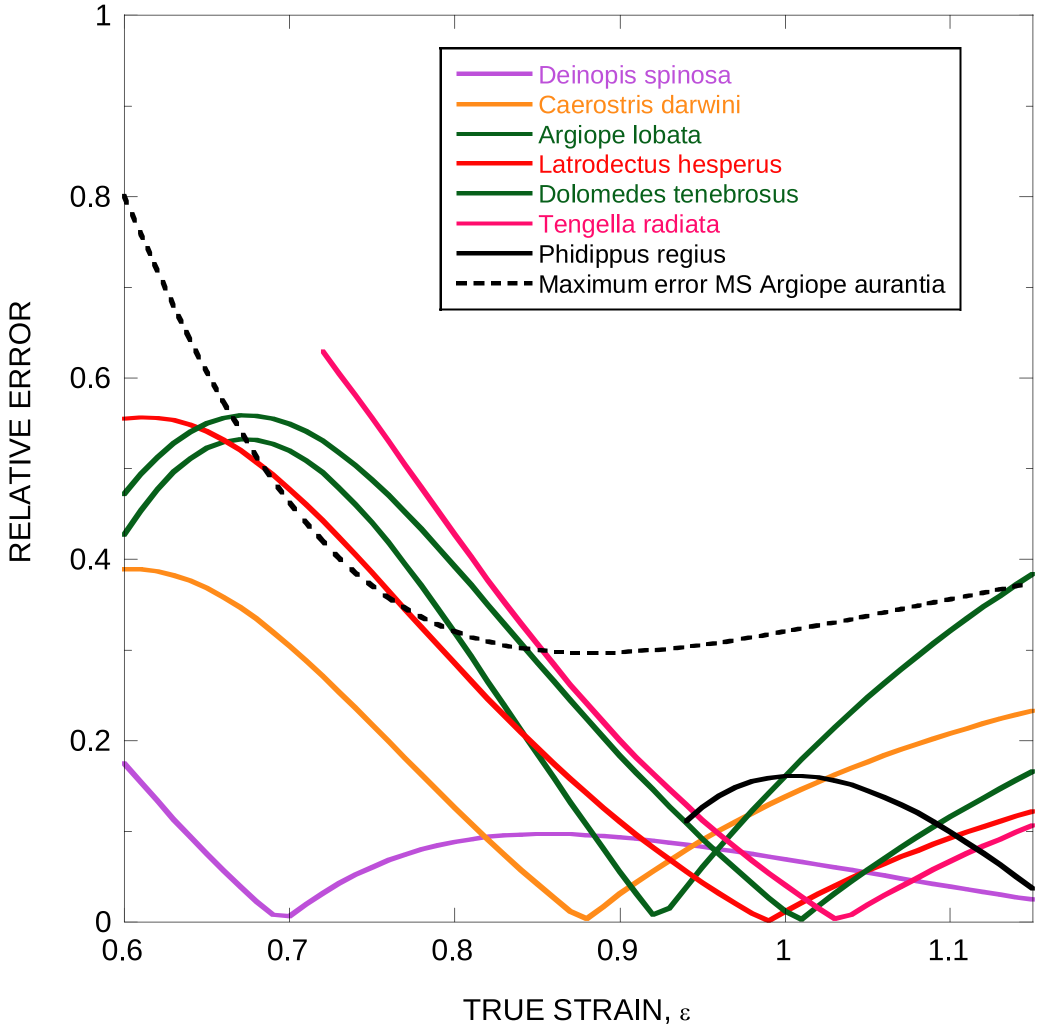

Supplementary Material Figure 2c

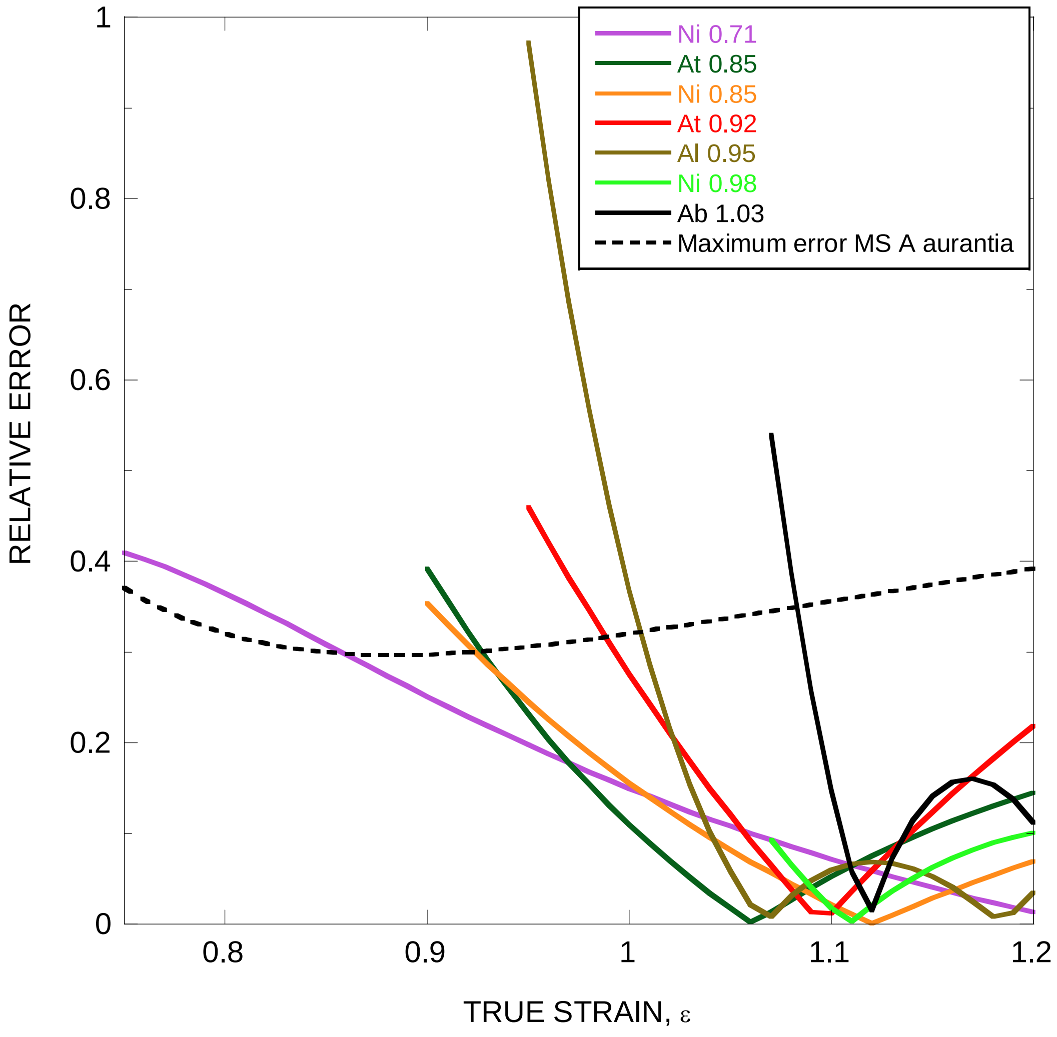

Supplementary Material Figure 2d

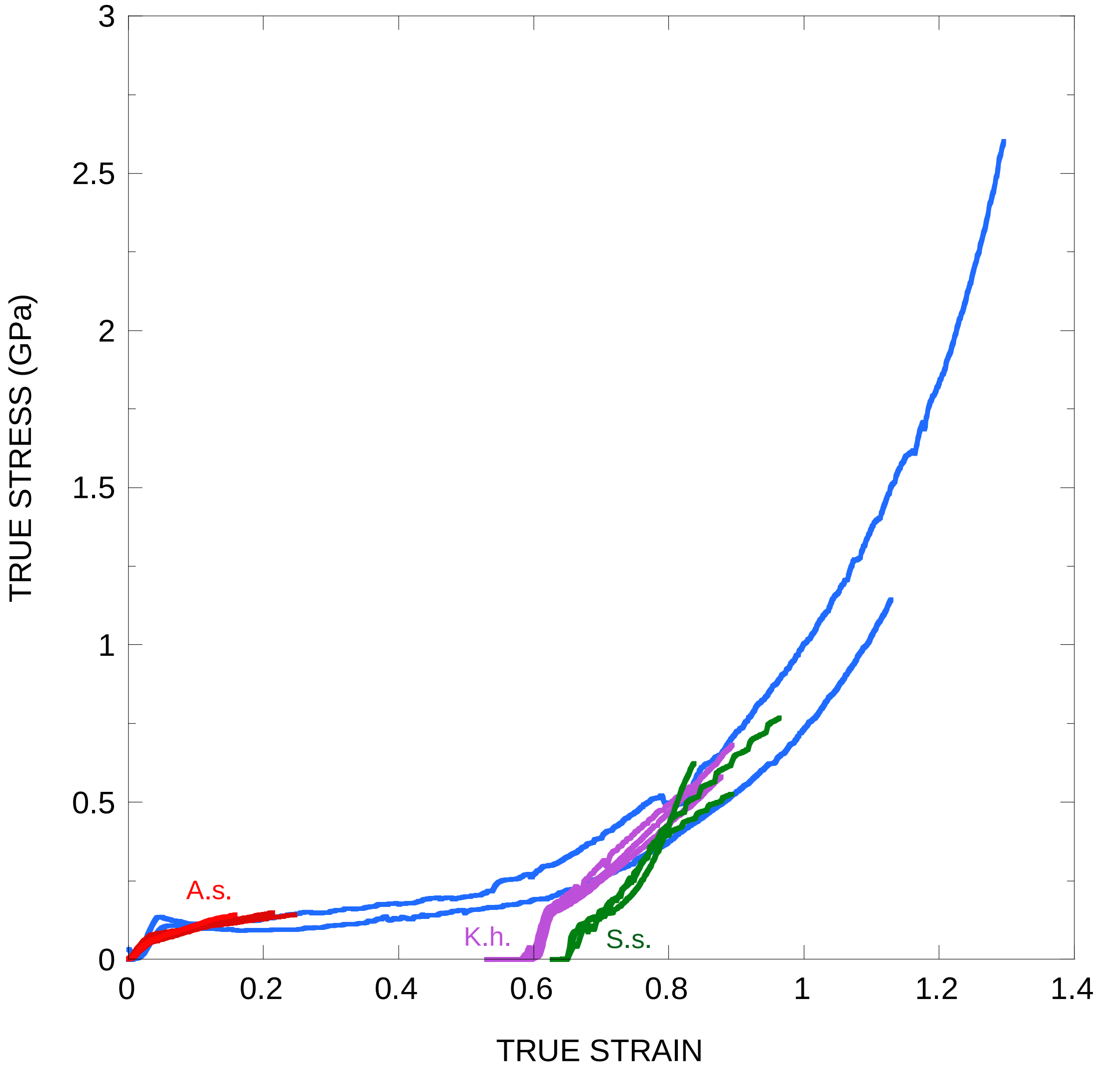

Supplementary Material Figure 3

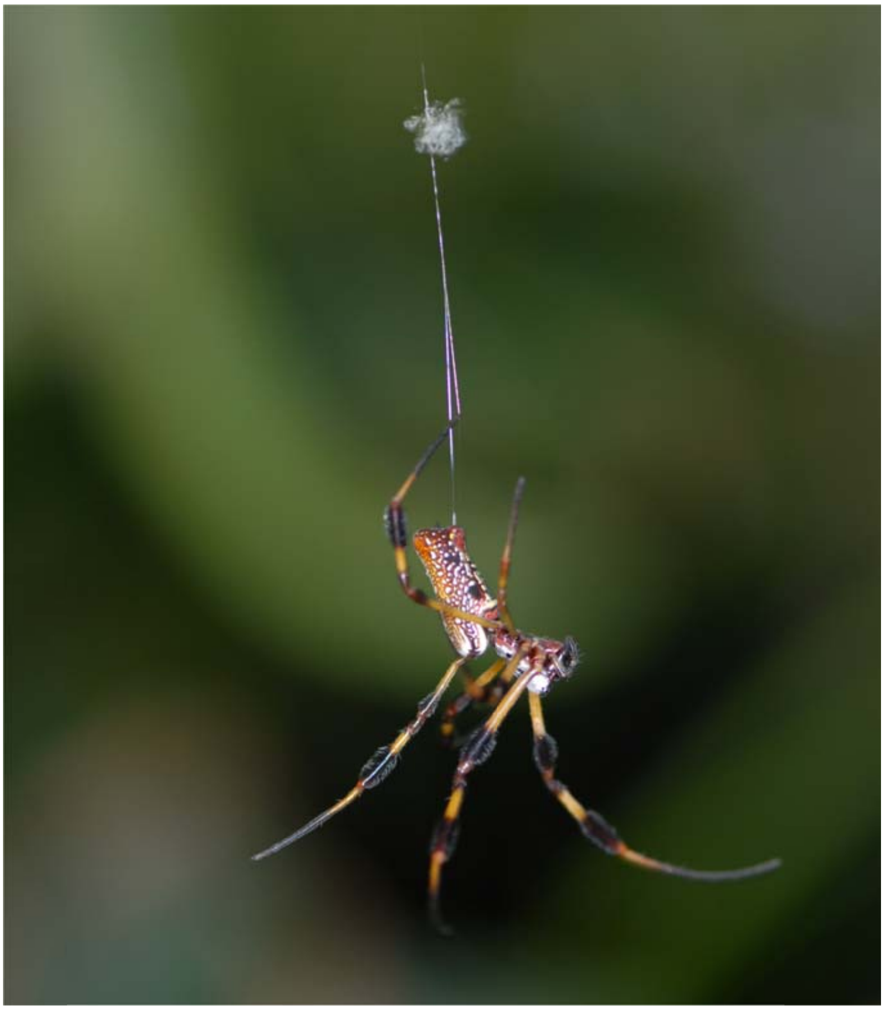

Supplementary Material Figure 4
